# Exit tunnel modulation as resistance mechanism of *S*. *aureus* erythromycin resistant mutant

**DOI:** 10.1038/s41598-019-48019-1

**Published:** 2019-08-07

**Authors:** Yehuda Halfon, Donna Matzov, Zohar Eyal, Anat Bashan, Ella Zimmerman, Jette Kjeldgaard, Hanne Ingmer, Ada Yonath

**Affiliations:** 1 The Weizmann Institute of Science, The Department of structural biology, Rehovot, 7610001 Israel; 2 National Food Institute, Technical University of Denmark, Kemitorvet, DK-2800, Kgs, Lyngby, Denmark; 3 Department of Veterinary and Animal Sciences, Faculty of Health and Medical Sciences, University of Copenhagen, 1870 Frederiksberg, Denmark

**Keywords:** Cryoelectron microscopy, RNA

## Abstract

The clinical use of the antibiotic erythromycin (ery) is hampered owing to the spread of resistance genes that are mostly mutating rRNA around the ery binding site at the entrance to the protein exit tunnel. Additional effective resistance mechanisms include deletion or insertion mutations in ribosomal protein uL22, which lead to alterations of the exit tunnel shape, located 16 Å away from the drug’s binding site. We determined the cryo-EM structures of the *Staphylococcus aureus* 70S ribosome, and its ery bound complex with a two amino acid deletion mutation in its ß hairpin loop, which grants the bacteria resistance to ery. The structures reveal that, although the binding of ery is stable, the movement of the flexible shorter uL22 loop towards the tunnel wall creates a wider path for nascent proteins, thus enabling bypass of the barrier formed by the drug. Moreover, upon drug binding, the tunnel widens further.

## Introduction

**Figure 1:**
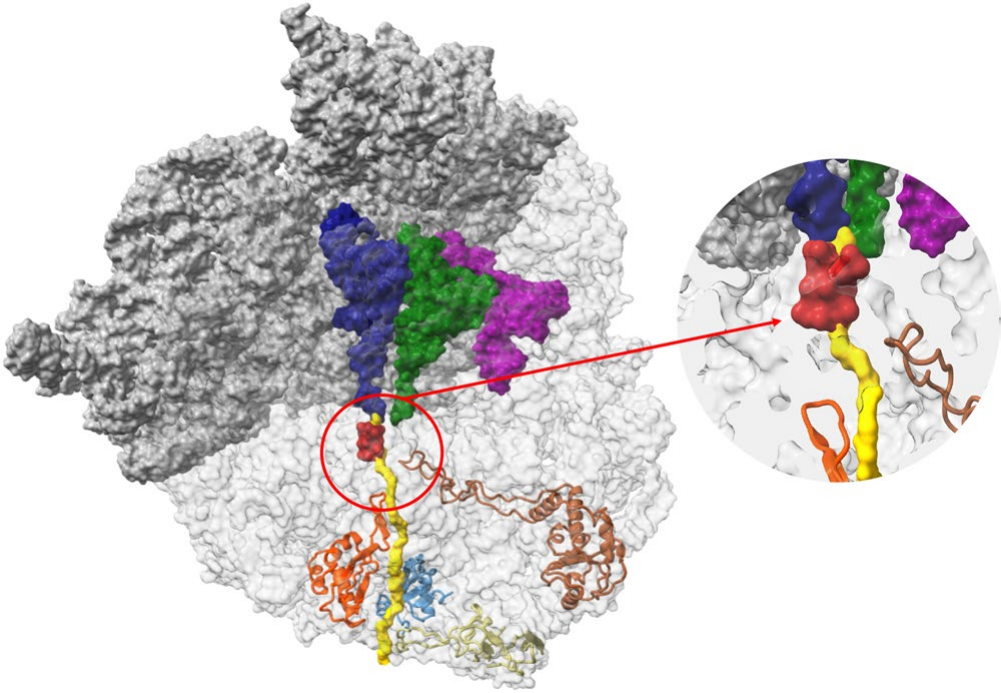
The ribosome nascent chain tunnel environment Left: The 70S *S*. *aureus* (SA_WT) ribosome where the large subunit is shown in light grey and the small subunit is shown in dark grey (PDBID 5TCU). The A-site, P-site and E-site docked tRNA molecules (from PDBID 5JTE) are shown in blue, green and magenta, respectively. The surface of a nascent chain within the tunnel is shown in yellow. Erythromycin surface is shown in red, uL4, uL22, uL23 and uL24 are shown in brown, orange, teal and khaki, respectively. Right: zoom into the ery binding site at the upper tunnel.

Several ribosomal antibiotics inhibit protein biosynthesis by targeting functional sites in the ribosome, including the decoding center, the peptidyl transferase center (PTC) and the ribosomal exit tunnel (NPET) in which the nascent proteins migrate until they emerge out of the ribosome. The NPET, which is lined mostly by the 23S ribosomal RNA (rRNA) chain, initiates near the PTC and transverses through the large ribosomal subunit (LSU). Four ribosomal proteins (rProteins), uL4, uL22, uL23 and uL24, of which the globular regions are located on the ribosome surface, extend to line the tunnel walls. Loops of uL4 and uL22 that form the tunnel narrowest constriction close to the tunnel entrance whereas uL23 and uL24, are part of the tunnel exit (Fig. 1). Previous studies showed that the tunnel interactions with specific sequence motifs of the nascent protein, which may lead to gene expression regulation due to translation arrest^1–13^.

Erythromycin (ery), the first macrolide antibiotic that was introduced clinically^14^, is composed of a 14-member macrolactone ring to which two sugar moieties, desosamine and cladinose are attached. It binds to the ribosome NPET wall at about 5–7 peptide bonds distance from the PTC (Fig. 1)^15^ and blocks part of the NPET, thus preventing the progretion of nascent peptide chains. It has been shown that for macrolides, the most common resistance mechanisms are by A2058G mutation (*E*. *coli* numbering is used throughout) at the antibiotic binding site or by the *erm* encoded methylase that methylates the exocyclic nitrogen of the adenine base^16–20^.

An additional resistance mechanism to ery was reported over 40 years ago in *E*. *coli* where a strain with three amino acids Met82-Lys-Arg deletion in uL22 showed high affinity to ery binding but was not inhibited by it^21–24^. Other bacteria and archaea, such as *H*. *marismortui*, *S*. *pneumoniae*, *S*. *aureus*, *S*. *pyogenes*, *H*. *pylori*, *H*. *influenza*, *F*. *tularensis* and *D*. *radiodurans* also exhibited similar behavior upon mutation in uL22^25–32^. The mutations in uL22 including insertions, deletions and single amino acid substitutions are mostly located on the ß hairpin loop of uL22, which extends from the globular domain of the protein and reaches the tunnel wall. Many of such mutated ribosomes maintain high affinity ery binding along with resistance to it^2,3,22,24^.

*S*. *aureus* (SA) is a gram-positive pathogen that is a major cause of infections acquired in hospitals and in the community, particularly if the cutaneous barrier has been damaged^3^. Many hospital-acquired infections are caused by highly resistant bacteria such as methicillin-resistant *S*. *aureus* (MRSA) and vancomycin-resistant *S*. *aureus* (VRSA)^33–35^ where both methicillin and vancomycin are targeting the cell wall rather than the ribosome. The high-resolution structures of the ribosome from *S*. *aureus* as well as of its complexes with a few clinically useful drugs and new potential inhibitors were determined in our lab^36^ and shed light on its explicit drug inhibition properties and selectivity as well as on its specific structural elements to be targeted. Directed evolution can be used for identifying and isolating mutated bacteria that show resistance to antibiotics and is a helpful tool for identifying new resistance mechanisms, which may lead to a better understanding of species-specific resistance mechanisms. It was applied for the isolation a uL22 mutant *S*. *aureus* ribosome (SAuL22m) from a wild type strain of *S*. *aureus*, which harbors a 2 amino acid deletion in the ß hairpin loop of uL22^26^.

We present here the single particle cryo-EM high resolution structures of the apo and ery bound SAuL22m mutant ribosome, which demonstrates how deletion of R88-A89 in *S*. *aureus* ribosomal protein uL22 ß hairpin loop leads to ery resistance although it does not hamper the binding of the antibiotic itself. We also show that upon ery binding additional conformational changes occur that are beyond the expected changes, occur as a result of the deletion per se. In addition, by comparing the current structures with the native SA ribosome and additional uL22 mutants structures from other bacterial species^17,25,26^, we highlight the specific structural changes that occurred in each of the uL22 mutant proteins which nevertheless led to a similar outcome at the cellular level.

## Results and Discussion

**Figure 2:**
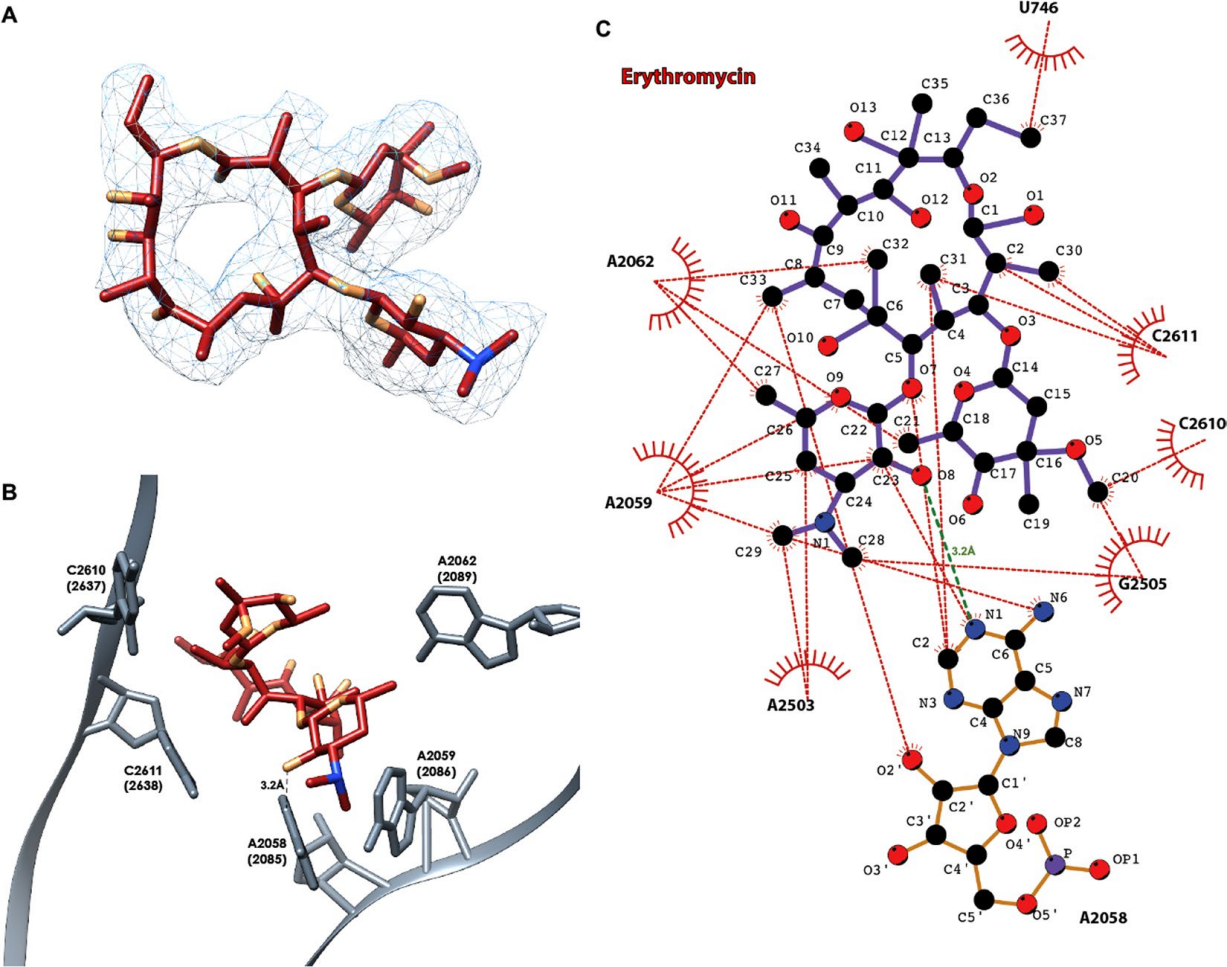
The binding pocket of erythromycin within SAL22m_ery structure. (**A**) Ery 3D structure as modeled in the cryo-EM map of the SAL22m_ery complex structure. The map is contoured around the ligand at 3σ. (**B**) Ery binding site in the SAuL22m_ery complex structure at the large subunit. Among the rRNA nucleotides that form the ery binding pocket, marked is the hydrogen bond between A2058 and ery (numbering according to *E*. *coli* with *S*. *aureus* numbering in parentheses). (**C**) Ery interactome within its binding pocket at the SAuL22m_ery ribosome. Ery maintains a dense array of electrostatic interactions with rRNA residues within the binding pocket at the LSU. rRNA nucleotides are numbered according to the *E*. *coli* numbering. Hydrogen bonds and hydrophobic interactions are presented as green and red dashed lines. Bond lengths are presented in ångström (Å).

The single particle cryo-EM structures of the 70S ribosomes from apo uL22 mutant *S*. *aureus* (SAuL22m_apo) and of its complex with ery (SAuL22m_ery) were determined at 3.58 Å and 3.2 Å, respectively (Table S1). A focused refinement of both large subunits resulted in a 2.4 Å and 2.3 Å cryo-EM reconstructed maps, respectively. These maps allowed for the unambiguous assignment of ery within SAuL22m_ery complex structure (Fig. 2A) and for defining the interactions of ery with the rRNA nucleotides at the binding site (Fig. 2B) including the identification of the nucleotides that bind ery via hydrophobic interactions as well as its hydrogen bonds (Fig. 2C). Ery inhibition assays using SA_WT and SAuL22m ribosomes clearly show that the mutant ribosomes are resistant to ery (Fig. S1).

**Figure 3:**
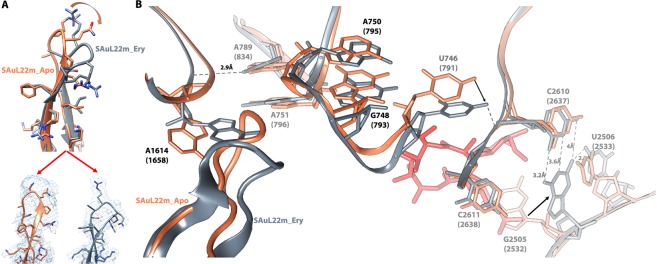
Conformational changes in the mutant upon erythromycin binding. (**A**) uL22 ß hairpin loop tip modeled into the cryo-EM map of SAuL22m_apo (coral) and of SAuL22m_ery (grey). (**B**) The cascade of movements of rRNA and rProteins that occur upon ery binding to the mutant ribosome.

**Figure 4:**
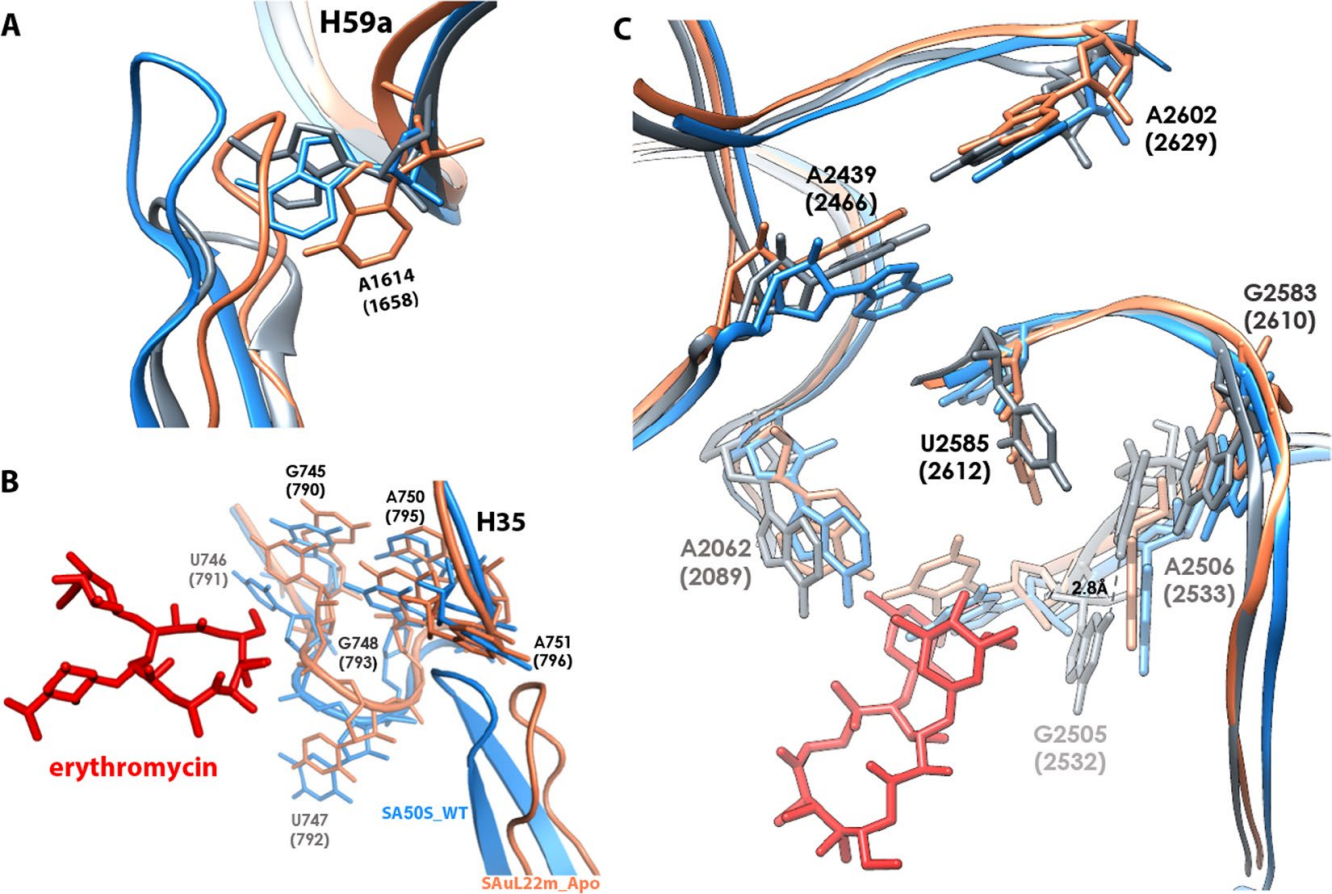
Comparison of uL22 ß hairpin loop and ery binding pocket in SA50S_WT vs. SAuL22m_apo and SAuL22m_ery. (**A**) Overlay of uL22 ß hairpin loop tip of SA50S_WT, SAuL22m_apo and SAuL22m_ery structures in blue, coral and grey, respectively. Nucleotide A1614 undergoes the largest movement within uL22 ß hairpin loop conformational changes. (**B**) H35 conformational changes upon mutation near uL22 ß hairpin loop; Overlay of SA50S_WT and SAuL22m_apo structures (blue and coral, SAuL22m_ery complex structure is not shown since it overlaps very well with SAuL22m_ery structure at this region) display a shift in H35 towards the uL22 loop new position in the mutant. (**C**) Overlay of SA50S_WT, SAuL22m_apo and SAuL22m_ery structures in blue, coral and grey, respectively, showing the ery binding pocket region, highlighting the rRNA nucleotides undergo conformational changes.

The cryo-EM maps allowed for the unambiguous tracing of the uL22 hairpin loop of both SAuL22m_apo and its complex structure with ery (Fig. 3A). Upon superposition of the SA50S wild type (PDBID 6HMA, SA50S_WT) and the SAuL22m_apo structures, we identified conformational changes of uL22 that are due to the deletion mutation that shifts the beta hairpin loop by 6A from its location in the wild type. Consequently, next to the uL22 ß hairpin loop the rRNA nucleotides A1614 (located in H59a tip) is rotated about 45° (Fig. 4A) and H35 is displaced by about ~3.0 Å (Fig. 4B).

**Figure 5:**
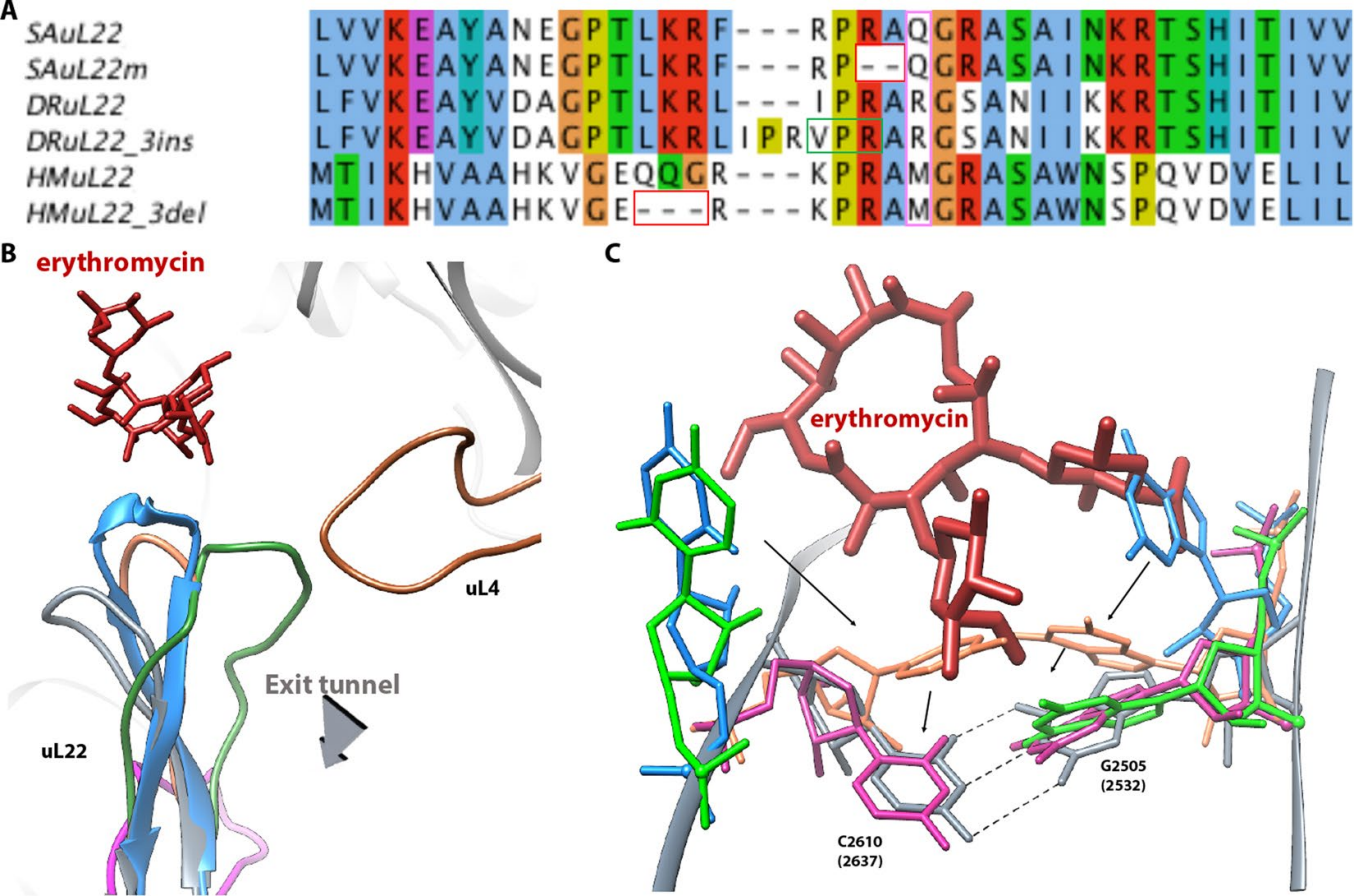
Comparative analysis of the uL22 ß hairpin loop and ery stabilization. (**A**) Sequence alignment of uL22 ß hairpin loop region (residues 69–107) from *S*. *aureus*, *D*. *radiodurans* and *H*. *marismortui* and their mutant counterparts. The sites of the deletions in the mutants are marked in red squares and the insertion site is marked by a green square. Residue 90 is marked by a purple box. (**B**) The superposition of uL22 ß hairpin loop from SA50S_WT (PDBID 5NGM blue), SAuL22m_apo (coral), SAuL22m_Ery (grey), DRuL22_3ins (PDBID 4WFN, green) and HMuL22_3del (PDBID 1YJ9, magenta) structures. Ery structure is shown in red. SAuL22m_apo loop has a similar conformation to the SA50S_WT (with a shorter loop due to deletion) while in SAuL22m_Ery complex structure the uL22 tip points away from the tunnel wall. (**C**) Upon ery binding to SAuL22m (grey), a new WC base pair, which stabilizes ery binding, is formed between rRNA nucleotides G2505 (2532) and C2610 (2637). An overlay of SA50S_WT (blue), SAuL22m_apo (coral), E70S (PDBID 4V7U, pink), D50S (PDBID 4WFN, green). The conformational changes of rRNA nucleotides C2610 and G2505 are shown. Between SA50S_WT (blue) and the mutant apo in SAuL22m_apo structure (coral), a movement of about ~45^o^ and ~90^o^ respectively is observed. Upon ery binding, the movement proceeds by ~35^o^ and ~90^o^ in SAuL22m_ery (grey) to form the WC base pairing. A similar BP has been observed in E70S (PDBID 4V7U) (pink) and in T70S (PDBID 4V7X). However, in *D*. *radiodurans* uL22 mutant (D50SL22m) no such movement has been observed (green).

In addition, we identified several conformational changes at the macrolide’s binding site of nucleotides A2062, A2439, G2505, U2585, G2583 and A2602. A small movement of A-site nucleotides and helix H92 was also detected. Some of these changes are significant, namely, A2439 shifts about 3 Å and U2585 is about 45° rotated (Fig. 4C). Nucleotides G2505 and C2610 are rotated by about 45° and 90° respectively (Fig. 5C).

By the superposition of the SAuL22m_ery complex structure on the two apo structures above we could identify several conformational changes that occurred upon ery binding; At the binding site, A2062 is 45° rotated, U2585 is 110° rotated, G2505 is 90° and U2506 is about 3.5 Å shifted in order to accomodate G2505 and forms a hydrogen bond with it (Fig. 4C). A small rotation of about 20° of nucleotide G2610 was also identified (Fig. 5C).

Interestingly, upon ery binding, G2505 is about 30° rotated towards C2610, which is 45° rotated, to form a WC base pair (BP) with it, where this movement seems to be at the final step in the mid-way movement shown in SAuL22m_apo relative to SA50S_WT. A similar BP was also identified in the structures of *E*. *coli* and *T*. *thermophilus* complexes with ery (PDBID 4V7U, EC70S and PDBID 4V7X, respectively); however, in both *D*. *radiodurans* uL22 mutant apo and ery bound structures (PDBID 4WFN and 4U67, respectively) no such movement has been identified. This movement stabilizes ery binding to the mutant by providing additional hydrophobic interaction (Fig. 5C).

By inspecting the structural changes that occur upon ery binding to the mutant ribosome we could identify a cascade of movements (Fig. 3B). At the ery binding site, nucleotide G2505 rotates almost 90°, forms a new base pair with C2610 and is stabilized by another bond with U2506. U746 moves together with H35 by about ~3.0 Å to form a bond with OP1 of C2611. Then, A1614 rotates by about 45^o^ where this rotation is stabilized by a bond between N6 from A789 and OP2 of A1614. Due to such cascade of events uL22 ß hairpin loop shifts to the tunnel wall.

By comparison of uL22 ß hairpin loop of the SA50S_WT, SAuL22m_apo and SAuL22m_ery structures, we found that the loop is shifted towards the center of the tunnel in the SAuL22m_apo structure whereas in SAuL22m_ery complex structure it is shifted back closer to its location in SA50S_WT structure (Fig. 4A). Nevertheless, the tip of the beta hairpin loop moves towards the tunnel wall and a new cavity that adds to the tunnel width forms (Figs 5B and S4).

These findings, combined with binding assays that showed no changes in the binding affinity of ery to native SA ribosome compared to the mutant ribosome^26^, are in line with the clear electron density of ery. Thus, it supports the idea that the uL22 mutation does not dramatically affect ery binding site, but provides an alternative mechanism for nascent proteins progression through the NPET.

Previous studies suggested that nascent proteins can bypass the ery in the ribosome by stabilizing A2062 in a conformation that increases the space available for their passage^37^. We add to this proposed stabilization of A2062, the deletion mutation at the tip of the ß hairpin loop that forms an additional free space in the tunnel through which the nascent proteins can bypass the antibiotic (Fig. 5). By comparing the uL22 hairpin region among SA50S_WT, EC70S (PDBID 4V7U) and SAuL22m_apo, a new groove was identified. Upon ery binding, an additional groove form in SAuL22m_ery structure. Thus, we suggest that the uL22m new groove widens the tunnel in which the nascent protein can pass and bypass ery’s steric blockage (Fig. S4). This mechanism, which is activated upon drug binding, is a new finding that suggests a rearrangement of the tunnel further to the expected changes due to the deletion mutation. It also supports the necessity to study the complex SAuL22m_ery structure.

Sequence alignment of other, similar, uL22 ery resistant mutant ribosomes from the archaea *H*. *marismortui* and the eubacteria *D*. *radiodurans*, indicates that the mutations occur in proximity to conserved positions around the tip of the beta hairpin loop (Fig. 5A) and the specific mutated nucleotides in the SAuL22m are highly conserved among bacteria. Our studies explain why changes in this region of uL22 are crucial for the destabilization of its loop and the development of the antibiotic’s resistance. Comparative structural studies of the various uL22 mutants’ ribosomes reveal different conformations of the loop. The structure of *H*. *marismortui* uL22m_del3 with no bound antibiotics (PDBID 1YJ9) shows that a three amino acids deletion further downstream of the ß hairpin loop leads to a change of the loop conformation from the tunnel wall which leads to a widening of the tunnel (Fig. 5B). The structure of a three amino acids insertion in *D*. *radiodurans* in complex with ery (PDBID 4WFN) displays a widening of the uL22 tip itself and reveals a small movement of the loop towards the tunnel wall (Fig. 5B). The importance of residue 90 of uL22, which is not conserved among bacterial species, for erythromycin resistance was recently reported^38^. This finding further supports our results since the deletion is in proximity to Q90 and changes its location.

**Figure 6:**
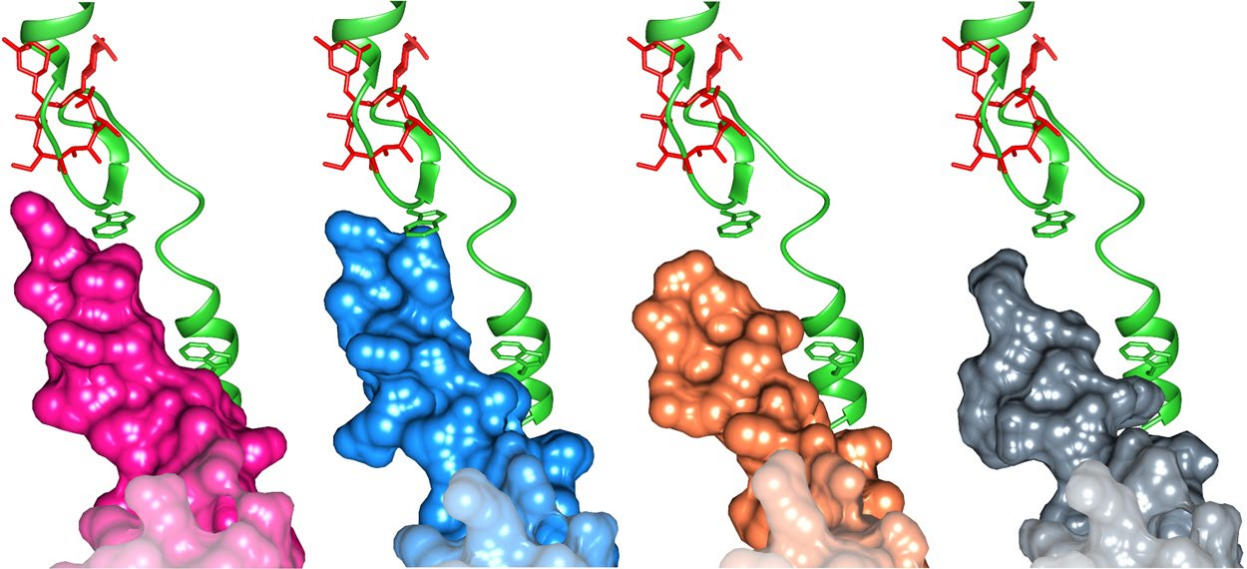
VemP interaction with uL22. A view of VemP (green) with a surface representation of uL22 from *E*. *coli* (green), SA50S_WT (blue) both shows a similar structure while SAuL22m_apo (coral) and SAuL22m_ery complex (grey) reveals a wider path made possible by the shortening of the ß hairpin loop and the additional grove upon ery binding to SAuL22m_ery. Ery position is shown in red.

A recent study reported that a Vibiro export monitoring polypeptide (VemP) acts as a *cis*-regulatory polypeptide and interacts with R92 and R95 of uL22 in order to stall the ribosome^13^. Upon superposition of VemP of *E*. *coli* (PDBID 5NWY) on SA50S_WT, SAuL22m_apo and SAuL22m_ery we observed that while in *S*. *aureus* uL22 residue 95 is alanine instead of arginine the overall structure remains the same in SA50S_WT. However, in mutant SAuL22m_apo there is a vast opening in proximity to the ß hairpin loop and the new groove lead to a more spacious tunnel for peptides movement through the tunnel. Upon ery binding to the mutant, in SAuL22m_ery structure, additional grooves are formed, which leads to a wider tunnel (Fig. 6). We suggest that this change affects resistance to ery while potentially preserving the stalling function of the *cis*-regulatory polypeptides, which are important to the normal function of the bacteria. A recent computational study^39^ suggests that erythromycin slows or stalls synthesis of ErmCL compared to H-NS due to stronger interactions with particular residue positions along the nascent protein. uL22 various mutations may change the rate of stalling of specific protein synthesis while ery is bound at the NPET and changes the electrostatic and dispersion interactions with nascent proteins.

Our results confirm that diverse mutations in the rProtein uL22 ß hairpin loop occur in various bacterial species and, by applying slightly different mechanisms, they facilitate nascent protein progression in the exit tunnel. All are overcoming the erythromycin binding at the upper tunnel wall, resulting in a seemingly common, however somewhat different, resistance mechanism against the drug at the bacterial level.

## Materials and Methods

### Ribosome purification

The bacteria were isolated and grown as described^26,36^. The bacteria were lysed enzymatically using lysostaphin (50 mg/ml) in 10 mM Hepes pH = 7.6, 30 mM MgCl_2_, 150 mM NH_4_Cl, 6 mM ß -mercaptoethanol for 45 minutes at 37 °C. The lysate was then centrifuged for 45 minutes at 20,000 rpm in Ti-70 rotor. The supernatant was layered on 1.1 M of sucrose cushion and centrifuge for 20 hours at 40,000 rpm in Ti-70 rotor. The supernatant was discarded, and the pellet was dissolved in 10 mM Hepes pH = 7.6, 14 mM MgCl_2_, 100 mM NH_4_Cl, 50 mM KCl, 6 mM ß -mercaptoethanol buffer. The 70 S ribosomes were purified by sucrose gradient ultracentrifugation, on a gradient of 10–50% sucrose in the same buffer for 17.5 hours at 18,000 rpm in SW-28 rotor. The samples were kept in 10 mM Hepes pH = 7.6, 10 mM MgCl_2_, 60 mM NH_4_Cl 15 mM KCl buffer and brought to a final concentration not exceeding 1,000 A260 mL^−1^, and then flash-frozen in liquid nitrogen and stored at −80 °C.

### Ribosomal inhibition assay

The inhibition assay was performed in 160 mM Hepes-KOH (pH 7.5), 6.5% PEG 8 K, 0.074 mg/ml tyrosine, 1.3 mM ATP, 0.86 mM CTP, GTP and UTP, 208 mM potassium glutamate, 83 mM creatine phosphate, 28 mM NH4OAc, 0.663 mM cAMP, 1.8 mM DTT, 0.036 mg/ml folinic acid, 0.174 mg/ml *E*.*coli* tRNA mix, 1 mM amino acid, 8 μM Mg(OAc)_2_, 0.25 mg/ml creatine kinase, 0.027 mg/ml T7 RNA polymerase 0.003 μg/μl luciferase plasmid and *E*.*coli* S100 lysate (which doesn’t include ribosomes) and added 300 nM of the ribosomes. A concentration range of erythromycin was 140μM-0.01 μM in 1:2 serial dilutions. The results were plotted and IC50 values were calculated using the program GraFit 7. IC50 values were determined by fitting the inhibition data to a four-parameters IC50 equation: = $\,\frac{range}{1+{(\frac{x}{IC50})}^{s}}$ where Range is the maximum y range, and s is a slope factor. The x axis represents the concentration of the analyte. Data fitted to this equation are usually displayed with a logarithmically scaled x axis. The visualization of the data is obtained using GraFit software^40^.

### Complex preparation

The SAuL22mery was peppered by incubating 23 μl of the 0.3 mg/ml ribosome in 10 mM Hepes pH = 7.6, 10 mM MgCl_2_, 60 mM NH_4_Cl 15 mM KCl buffer for 30 minutes at 26 °C, then 1.1 μl of 10 mM ery dissolved in 10% Ethanol was added and the mixture was incubated for 20 minutes on ice.

### EM Sample preparation

Both samples were flash frozen using Vitrobot^TM^ VI (FEI) using the following conditions: For the SAuL22m_apo grids a ribosome concentration of 1 mg/ml was used on QUANTIFOIL® R 1.2/1.3. while for SAuL22M_ery grids, a ribosome concentration of 0.3 mg/ml was used on QUANTIFOIL® R 2/2 grids with continues carbon support.

### Data collection, processing and refinement

The cryo-EM data for both SAuL22m_apo and SAuL22m_ery structures were collected at the ESRF CM01 beamline^41^ using FEI Titan Krios (FEI) with K2 Summit (Gatan) direct electron detector, and Quantum LS imaging filter (Gatan) at a magnification of x130K, at defocus range of −0.5–1.0 nm and Pixel size of 1.067 A. The cryo-EM data for SAuL22m_apo structure were collected at 6.367 e^−^/pix/s and 5.227 e^−^/A2/s. 40 frames per micrograph of 8 sec total length, 0.2 sec per frame and a total dose of 40 e^−^/A2. 3542 movies were collected. From these micrographs 529,786 particles of SAuL22m_apo were selected for the 2D classification, from them 145,897 particles were selected for 3D classification and 124,731 particles were used for the 3D refinement which gave a 3.58 Å resolution map for the whole 70 S ribosome and a resolution of 3.2 Å resolution map for the 50 S ribosomal LSU (Fig. S2, Table S1).

The cryo-EM data for SAuL22m_ery complex structure were collected at 5.231 e^−^/pix/s and 4.288 e^−^/A2/s. 28 frames per micrograph of 7 sec with total length of 0.25 sec per frame and total dose of 30 e^−^/A2. 4161 movies were collected. From these micrographs 734,247 particles of SAuL22m_ery were selected for the 2D classification, from them 426,250 particles were selected for 3D classification and 378,309 particles were used for the 3D refinement which gave a 2.42 Å resolution map for the whole 70 S ribosome and a resolution of 2.3 Å resolution map for the 50 S ribosomal LSU (Fig. S2, Table S1).

Data processing of SAuL22m_apo was performed using Relion 2.1. Whereas data of SAuL22m_ery were processed using Relion 3.0-beta-2^42^. The PDBID 5NGM was used as a starting model for the modeling of SAuL22m_apo structure whereas the model of SAuL22m_apo was subsequently used as an initial model for SAuL22m_ery starting model. Manual refinement and modeling were done using Coot^43^ and refinement and validation were done using PHENIX^44^. Final validation and scoring were performed using MolProbity^45–47^.

### Figure generation and sequence alignment

All figures were generated with Chimera and ChimeraX^48,49^. Ribosome-erythromycin binding plot was generated using LigPlot+^50^. uL22 multiple sequence alignment was performed by ClustalW^51^ and presented by Jalview^52^.

### Accession numbers

Atomic coordinates and structure factors for the reported EM structures have been deposited with the Protein Data bank under accession number 6S0X, 6S0Z, 6S12 and 6S13.

The EMDB accession number are 10076, 10077, 10078 and 10079.

## Supplementary information

Exit tunnel modulation as resistance mechanism of S. aureus erythromycin resistant mutant – supplementary materials
