# Supplementary material for: Exit tunnel modulation as resistance mechanism of S. aureus erythromycin resistant mutant
Source: Sci Rep. 2019 Aug 7;9:11460. doi: 10.1038/s41598-019-48019-1 (PMC6685948; doi:10.1038/s41598-019-48019-1)
Supplement: Supplementary file 1 — Exit tunnel modulation as resistance mechanism of S. aureus erythromycin resistant mutant – supplementary materials [file 41598_2019_48019_MOESM1_ESM.docx]

**Exit tunnel modu­lation as resistance mechanism of *S. aureus* erythromycin resistant mutant**

Yehuda Halfon^1^, Donna Matzov^1^, Zohar Eyal^1^, Anat Bashan^1^, Ella Zimmerman^1^, Jette Kjeldgaard^2^, Hanne Ingmer^3^, Ada Yonath^1^

^1^The Weizmann Institute of Science, The Department of structural biology, 234 Herzel St. Rehovot, 7610001, Israel.

^2^National Food Institute, Technical University of Denmark, Kemitorvet, DK-2800, Kgs. Lyngby, Denmark.

^3^Department of Veterinary and Animal Sciences, Faculty of Health and Medical Sciences, University of Copenhagen, Stigbøjlen 4, 1870 Frederiksberg, DK

**corresponding author**: Ada Yonath, ada.yonath@weizmann.ac.il Tel: +97289343028


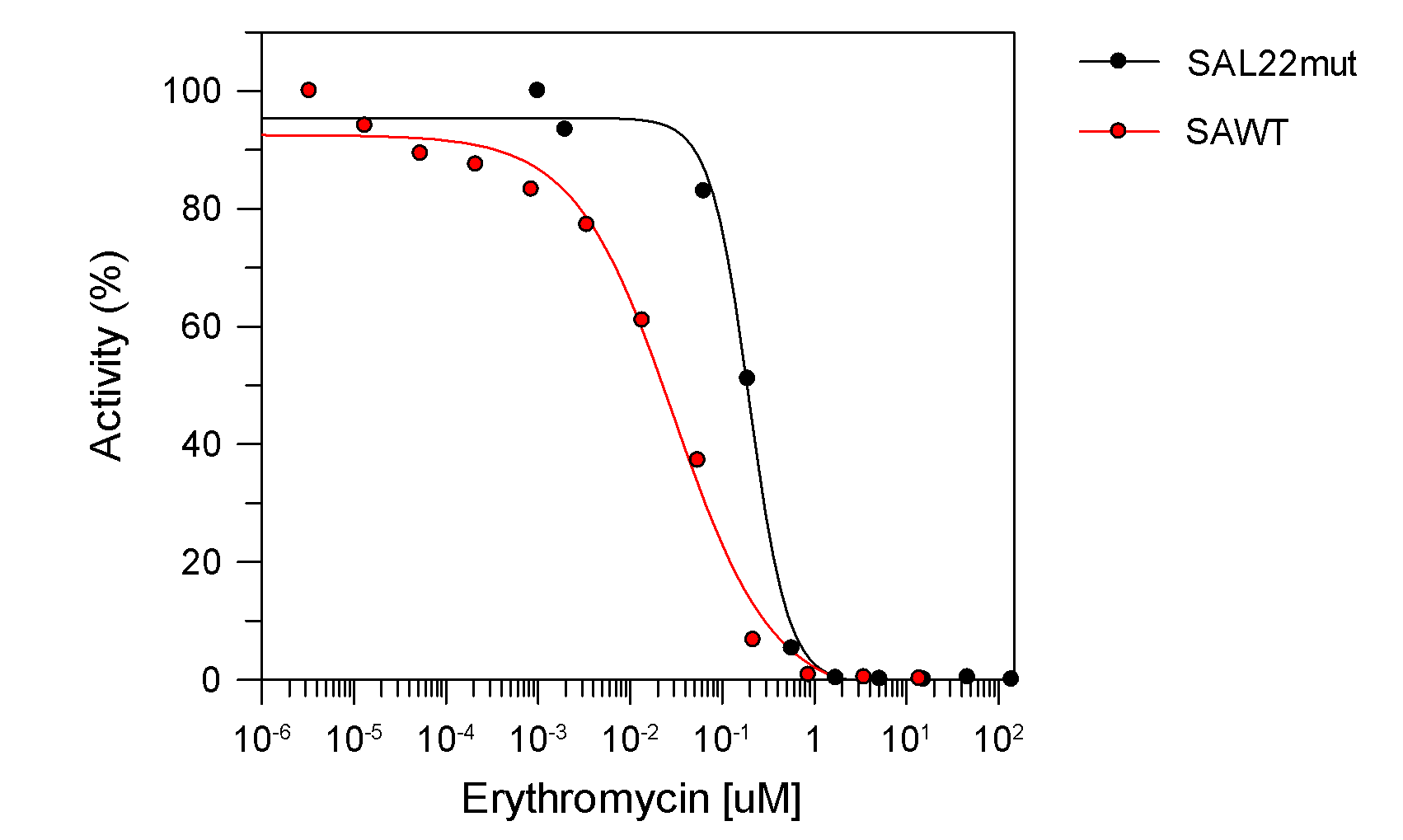


**Fig S1**:

**Inhibition of bacterial protein synthesis.** The inhibitory effect of erythromycin on protein expression in SA_wt (red) and SAuL22m (Black) *in vitro* transcription-translation cell-free system ^1^. The activity of the reporter protein (luciferase) in the presence of various concentrations of erythromycin is shown as an arbitrary unit of luminescence [a.u.]. The IC50 values calculated by the plotted data are 0.0298μM and 0.1963μM for SA WT and SAuL22m, respectively.


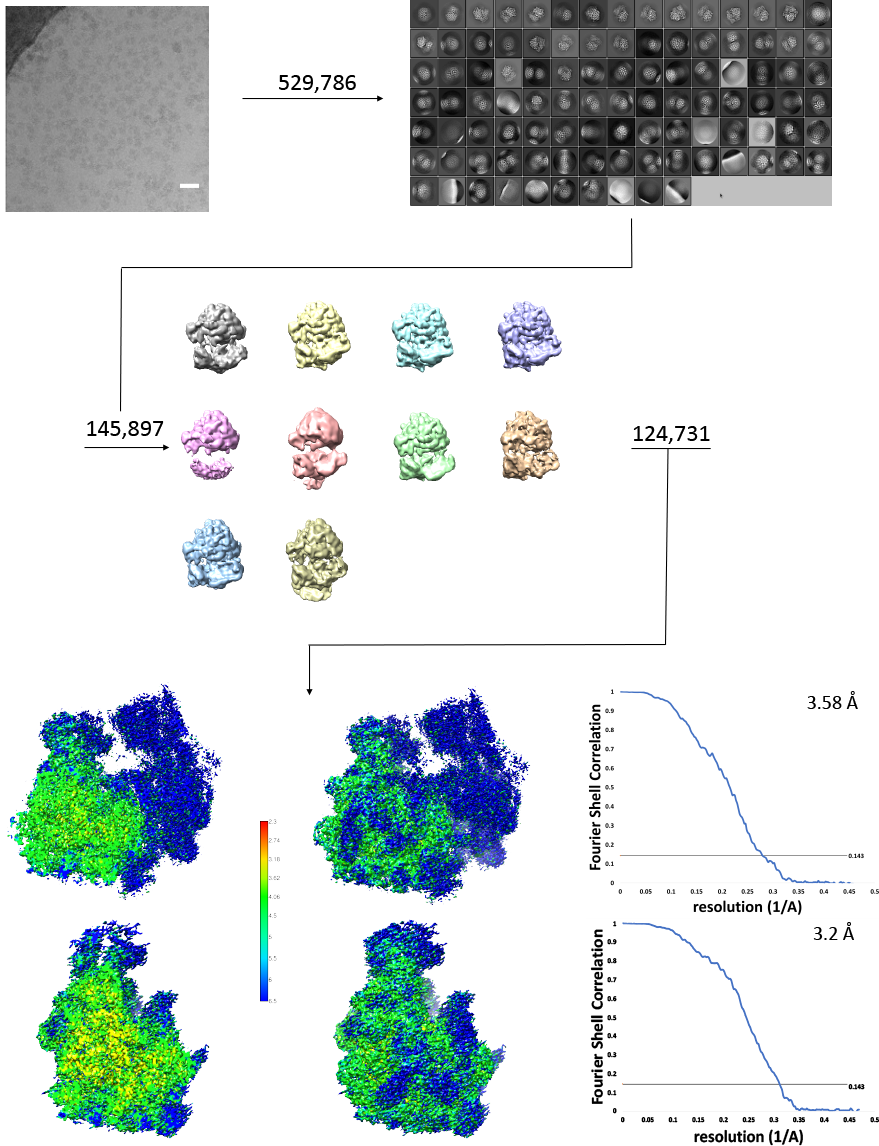


**Fig S2**: **The data processing workflow of SAuL22m_apo cryo-EM maps**. A representative cryo-EM micrograph showing 70S ribosome particle distribution on an EM grid (scale bar, 40nm); Representative 2D class averages showing well-defined densities for the various particles. 3D class averages of 70S ribosome particles; Surface (left) and cross-section (right) rendering of the cryo-EM density maps colored according to local resolution of the 70S map and the 50S; “Gold standard” FSC curves (FSC=0.143) for the final 3D map of whole, indicates nominal resolutions at 3.58 Å while the 3D map of the LSU indicates nominal resolutions at 3.2 Å. The numbers above the arrows are of particles that were selected for the next step of the processing.


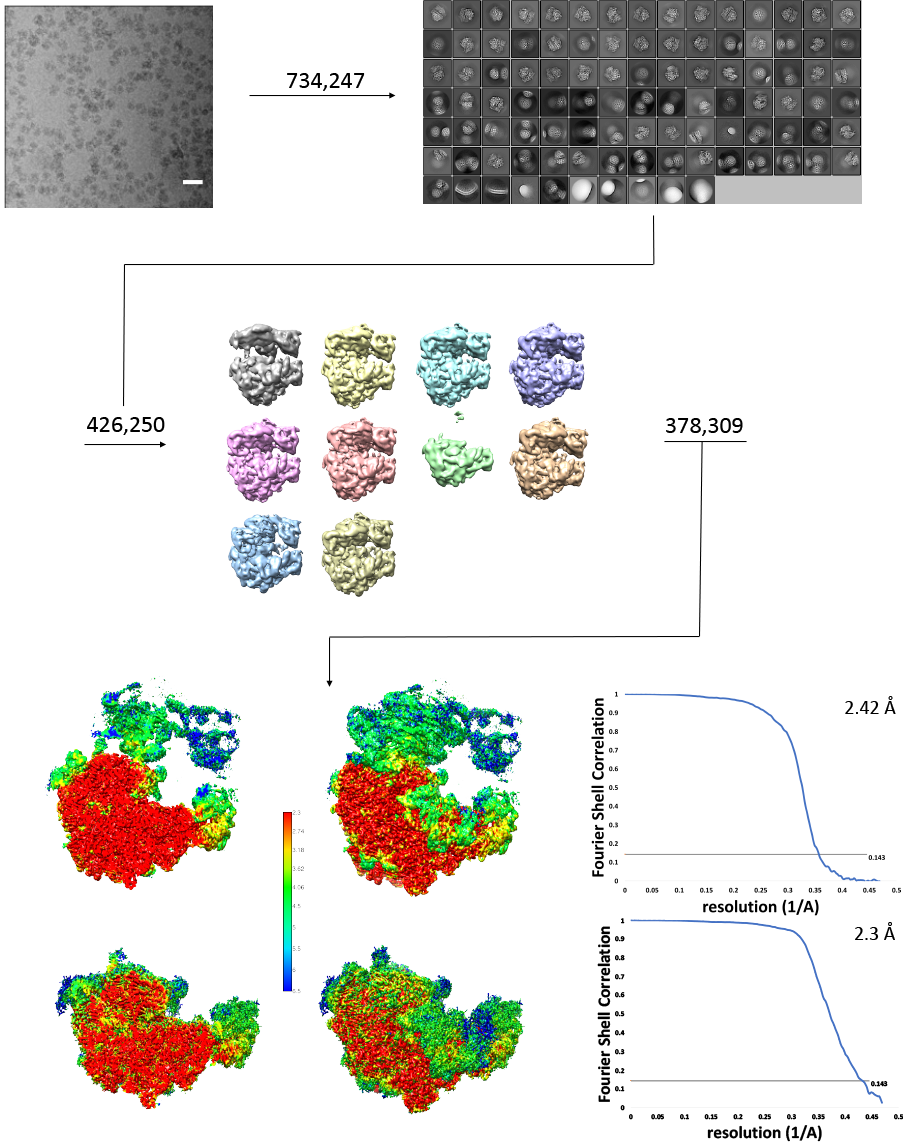


**Fig S3**: **The data processing workflow of SAuL22m_ery cryo-EM maps**. A representative cryo-EM micrograph showing 70S ribosome particle distribution on an EM grid (scale bar, 40nm); Representative 2D class averages showing well-defined densities for the various particles. 3D class averages of 70S ribosome particles; Surface (left) and cross-section (right) rendering of the cryo-EM density maps colored according to local resolution of the 70S map and the 50S; “Gold standard” FSC curves (FSC=0.143), for the final 3D map of whole, indicates nominal resolutions at 2.42 Å while the 3D map of the LSU indicates nominal resolutions at 2.3 Å. The numbers above the arrows are of particles that were selected for the next step of the processing.


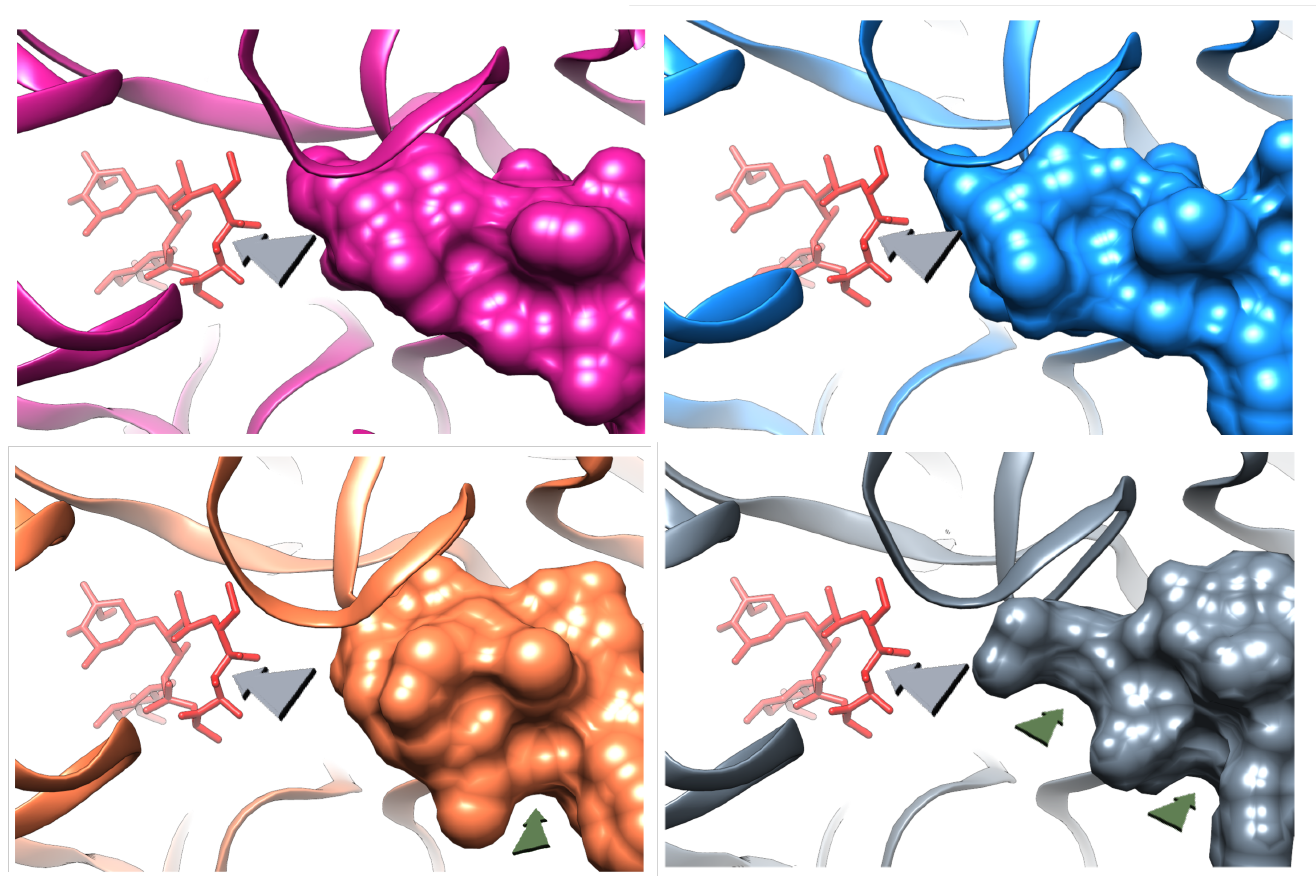


**Figure S4. uL22 ß hairpin loop tip at the ribosomal tunnel.**

A view to the NPET, from its internal side, showing the surface representation of uL22 ß hairpin loop and ery bound (grey arrow marks the NPET direction). EC50S (pink) and SA50S_WT (blue). Superficially, they look similar, but the loop is shorter in the mutant SAuL22m_apo structure (coral). A new groove is formed upon ery binding in SAuL22m_ery complex structure (grey). The green arrows point at the new groves.

**Table S1|** Cryo-EM data collection and model refinement

| **Data collection** | | | | |
| --- | --- | --- | --- | --- |
| Microscope | Titan Krios | | | |
| Camera | CMOS (K2 summit) | | | |
| Voltage (kV) | 300 | | | |
| Magnification | 135,000 | | | |
| Pixel size (Å.px^-1^) | 1.067 | | | |
| Name | SAuL22m_apo | | SAuL22m_ery | |
| Defocus range (µm) | 0.5-1.5 | | 0.5-1.5 | |
| Total dose (e/Å^2^) | 40 | | 30.016 | |
| Dose per frame | 1 | | 1.072 | |
| Micrographs collected | 3,542 | | 4,161 | |
| **Refinement** | | | | |
| Number of particles (autopicked) | 588,718 | | 734,247 | |
| Number of particles (used for 3D reconstruction) | 145,897 | | 378309 | |
| Subunit | 50S | 70S | 50S | 70S |
| Resolution (Å; at FSC^a^ = 0.143) | 3.2 | 3.58 | 2.3 | 2.42 |
| CC^a^ (model to map fit) | 0.83 | 0.8 | 0.85 | 0.55 |
| **RMS^a^ deviation** | | | | |
| Bonds (˚) | 0.02 | 0.04 | 0.01 | 0.05 |
| Angles (˚) | 0.02 | 0.04 | 0.04 | 0.21 |
| Chirality (˚) | 0.05 | 0.06 | 0.06 | 0.09 |
| Planarity (˚) | 0.007 | 0.006 | 0.004 | 0.006 |
| **Validation**^d^ | | | | |
| Clashscore^e^ | 12.37 | 27.51 | 2.91 | 9.09 |
| **Proteins** | | | | |
| MolProbity score | 3.07 | 3.68 | 2.18 | 2.98 |
| Rotamers outliers (%) | 33.81 | 21.31 | 7.85 | 16.28 |
| Ramachandran favored (%) | 91.23 | 79.39 | 92.82 | 88.97 |
| Ramachandran allowed (%) | 8.67 | 20.52 | 7.45 | 11.07 |
| Ramachandran outliers (%) | 0.1 | 0.09 | 0.03 | 0.02 |
| **RNA** | | | | |
| Correct sugar pucker (%) | 99.44 | 98.71 | 99.24 | 97.07 |
| Correct backbone conformation | 66.63 | 52.05 | 76.55 | 55.41 |
| ^a^FSC, Fourier shell correlation; CC, correlation coefficient; RMS, root-mean square.  ^b^Only across atoms in the model; compiled using Phenix.  ^c^Across whole map volume; compiled using Phenix.  ^d^Compiled using MolProbity.  ^e^Clashscore is the number of serious steric overlaps (>0.4 Å) per 1000 atoms. | | | | |

1 Murray, R. W., Melchior, E. P., Hagadorn, J. C. & Marotti, K. R. Staphylococcus aureus cell extract transcription-translation assay: firefly luciferase reporter system for evaluating protein translation inhibitors. *Antimicrob Agents Chemother* **45**, 1900-1904, doi:10.1128/aac.45.6.1900-1904.2001 (2001).
